# Altered regional brain activity and functional connectivity in resting-state brain networks associated with psychological erectile dysfunction

**DOI:** 10.3389/fnins.2023.1074327

**Published:** 2023-06-08

**Authors:** Xue Liu, Shaowei Liu, Tao Liu, Liang Tang, Mufan Ji, Yan Xu, Ziliang Xiang, Jianwen Zhou, Yun Chen, Jianhuai Chen

**Affiliations:** ^1^Department of Andrology, Jiangsu Province Hospital of Chinese Medicine, Affiliated Hospital of Nanjing University of Chinese Medicine, Nanjing, China; ^2^Department of Radiology, Jiangsu Province Hospital of Chinese Medicine, Affiliated Hospital of Nanjing University of Chinese Medicine, Nanjing, China; ^3^Medical College of Nantong University, Nantong, China

**Keywords:** psychological erectile dysfunction, resting state, functional magnetic resonance imaging, fractional amplitude of low-frequency fluctuation, functional connectivity

## Abstract

**Introduction:**

Erectile dysfunction (ED), especially psychological ED (pED), is usually accompanied with psychological factors, which are related to abnormal activity in brain regions involved in sexual behavior. However, the mechanisms underlying functional changes in the brain of pED are still unclear. The present study aimed to explore the abnormalities of brain function, as well as their relationships with sexual behavior and emotion in pED patients.

**Materials and methods:**

Resting state functional magnetic resonance imaging (rs-fMRI) data were collected from 31 pED patients to 31 healthy controls (HCs). The values of amplitude of fractional amplitude of low-frequency fluctuation (fALFF) and functional connectivity (FC) were calculated and compared between groups. In addition, the associations between abnormal brain regions and clinical features were evaluated by *Pearson* correlation analyses.

**Results:**

Compared to HCs, pED patients demonstrated decreased fALFF values in the left medial superior frontal gyrus (had decreased FC values with the left dorsolateral superior frontal gyrus), the left lingual gyrus (had decreased FC values with the left parahippocamal gyrus and insula), the left putamen (had decreased FC values with the right caudate) and the right putamen (had decreased FC values with the left putamen and the right caudate). The fALFF values of the left medial superior frontal gyrus were negatively correlated with the fifth item scores of International Index of Erectile Function (IIEF-5). Negative relationships were found between fALFF values of the left putamen and the second item scores of Arizona Sexual Scale (ASEX). FC values between the right putamen and caudate were negatively related to the state scores of State-Trait Anxiety Inventory (STAI-S).

**Conclusion:**

Altered brain function were found in the medial superior frontal gyrus and caudate-putamen of pED patients, which were associated with sexual function and psychological condition. These findings provided new insights into the central pathological mechanisms of pED.

## 1. Introduction

Erectile dysfunction (ED) is a common male sexual dysfunction, which manifests as inability to attain or maintain sufficient erection for satisfactory sexual intercourse. The occurrence of ED is considered to be associated with organic, psychological or mixed factors (Domes et al., 2021). Previous epidemiology studies had shown that the incidence rate of ED was positively correlated with age, especially for elderly individuals, and the prevalence rate in individuals aged 40–70 years was 52% (Feldman et al., 1994; Lewis et al., 2004). In 85.2% ED patients under 40 years of age, psychogenic factors were regarded as the primary cause, while 14.8% patients had organic factors as the primary causes (arterial, venous, neurological, endocrine, pharmacological, or mixed) (Nguyen et al., 2017). Psychological ED (pED) is the most common subtype of ED, which is owed predominantly to psychologic factors (Rosen, 2001). It was previously believed that pED was caused by psychological or interpersonal factors, such as anxiety, depression, lack of confidence or other psychological factors (Rosen, 2001; Bodie et al., 2003; Rosen et al., 2004). The diagnosis of ED is usually based on the sexual function scales, physical examination, nocturnal penile erection test, intracavernosal injection combined with color duplex doppler ultrasonography (ICI + CDDU) and laboratory examination (Hatzimouratidis et al., 2010; Hackett et al., 2018; Ravikanth, 2020; Domes et al., 2021). However, there is still little advance in the diagnosis of pED due to the lack of specific and objective biomarkers (Yin et al., 2019).

Functional magnetic resonance imaging (fMRI) has been widely applied to explore the central neural mechanisms underlying human sexual behavior (Mouras et al., 2008; Qin et al., 2015; Chen et al., 2016; Mills-Finnerty et al., 2022). A meta-analysis study demonstrated that the sexual arousal was associated with a lot of brain regions, including prefrontal, cingulate and parietal cortex, amygdala, thalamus, insula, caudate, putamen and hypothalamus (Stoléru et al., 2012). Based on resting-state fMRI (rs-fMRI), the central neural mechanisms of pED have been intensively explored. Previous study found that the occurrence of pED was related to abnormal functional connectivity (FC) between the left dorsolateral superior frontal gyrus and angular gyrus, as well as FC between posterior cingulate cortex and precuneus (Yin et al., 2020). In addition, gray matter atrophy was found in several subcortical structures such as amygdala, thalamus, hippocampus, caudate, putamen, pallidum, nucleus accumbens, and hypothalamus in pED patients (Cera et al., 2012). Moreover, damaged white matter in the prefrontal and limbic cortex also played a key role in the development of pED (Chen et al., 2018).

The measures of amplitude of low-frequency fluctuation (ALFF) and FC are common measures of rs-fMRI, which reflect the intensity of spontaneous activity in brain regions and the coactivation patterns of neurons between brain regions, respectively. The measure of ALFF had been applied to explore the central pathogenesis of pED and significantly lower baseline brain activity in the right orbitofrontal cortex and anterior insula was found to be associated with pE (Yin et al., 2020). However, ALFF is sensitive to physiological noise irrelevant to brain function. Therefore, fractional amplitude of low-frequency fluctuation (fALFF) was proposed to overcome this disadvantage in 2008 (Zou et al., 2008), which provided a more accurate low-frequency oscillation and a more effective response to the autonomous neural activity, as well as physiological state of specific brain regions (Zuo et al., 2010).

In this study, we aimed to explore the abnormalities of brain function by the measures of fALFF and FC, as well as their relationships with sexual function and emotion in pED patients. We hypothesized that altered brain function might be involved in the central pathological mechanisms of pED and related to the clinical features including ED and abnormal psychosocial status. Firstly, differences of brain activity between pED and healthy controls (HCs) were compared with the measure of fALFF. Secondly, brain areas with abnormal fALFF values were selected as regions of interest (ROI), and FC values between ROIs and the whole brain regions were calculated and compared between groups. Finally, we also evaluated the relationships between abnormal brain regions and the scores of International Index of Erectile Function (IIEF-5), Arizona Sexual Scale (ASEX), and State-Trait Anxiety Inventory (STAI-S).

## 2. Materials and methods

### 2.1. Participants

A total of 31 pED patients were included from the Department of Andrology, Jiangsu Province Hospital of Chinese Medicine, Affiliated Hospital of Nanjing University of Chinese Medicine. In addition, 31 right-handed, age and education level matched HCs were enrolled. The erectile function was assessed using IIEF-5 and ASEX while the level of anxiety was assessed using STAI by a clinician with more than 5 years of clinical experience.

Inclusion criteria for all subjects were as follows: (1) Han nationality; (2) right-handed; (3) aged from 20 to 45 years; (4) education level more than 9 years; (5) in a stable heterosexual relationship for more than 6 months; (6) had normal sexual desire; (7) had regular sexual intercourse. ED patients were diagnosed with IIEF-5 scores ≤ 21 and met the diagnostic criteria for ED as determined by the fifth edition of diagnostic and statistical manual of mental disorders (DSM-5): (i) persistent or recurrent inability to attain, or to maintain for satisfactory sexual intercourse; (ii) causes marked distress or interpersonal difficulty; (iii) is not better accounted for by another axis one disorder and is not due exclusively to direct physiological effects of substance abuse or general medical conditions (Segraves, 2010). Moreover, IIEF-5 scores of HCs were than more 21.

Exclusion criteria for all subjects were as follows: (1) combined with other sexual dysfunction; (2) partners with sexual dysfunction; (3) organic abnormalities, such as external genital trauma or congenital malformation, or obvious abnormalities in bilateral testes, epididymis and spermatic cord on palpation; (4) serious systemic diseases, such as cardiovascular, hepatic, renal, endocrine, hematopoietic, and neurological diseases; (5) history of psychoactive substance abuse and long-term use of antipsychotic drugs; (6) contraindication to MRI.

This study was approved by the Ethics Committee of Jiangsu Province Hospital of Chinese Medicine, Affiliated Hospital of Nanjing University of Chinese Medicine. All subjects were informed of the experiment before participating and signed informed consents.

### 2.2. MRI data acquisition and preprocessing

Magnetic resonance imaging (MRI) data were acquired using a 3.0T Siemens scanner. T1-weighted images were obtained with the following scanning parameters: repetition time (TR) = 1,900 ms, echo time (TE) = 2.48 ms, flip angle (FA) = 9°, number of slices = 176, slice thickness = 1 mm, field of view (FOV) = 250 × 250 mm^2^, matrix size = 256 × 256. Resting-state functional images were scanned interleaved with the following parameters: TR = 3,000 ms, TE = 40 ms, FA = 90°, number of slices = 32, slice thickness = 4 mm, slice gap = 0 mm, FOV = 240 × 240 mm^2^, matrix size = 64 × 64. The details about scanning parameters were presented in our previous studies (Chen et al., 2021; Liu S. et al., 2021; Xu et al., 2021).

**FIGURE 1:**
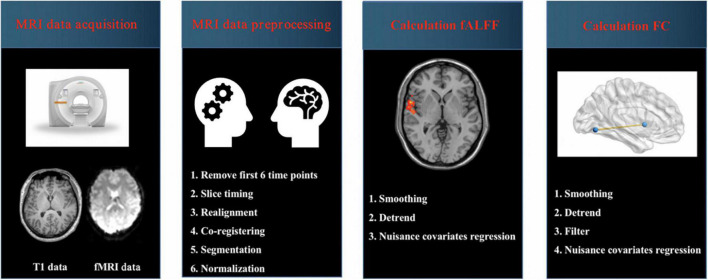
Schematic overview of functional magnetic resonance imaging (fMRI) data acquisition and processing. MRI, magnetic resonance imaging; fALFF, fractional amplitude of low-frequency fluctuation; FC, functional connectivity.

Magnetic resonance imaging (MRI) data were preprocessed by using Data Processing and Analysis for Brain Imaging (DPABI) software running in MATLAB (Yan et al., 2016). The steps of MRI data preprocessing were as follows: (1) data format conversion (DICOM to NIFTI); (2) removal of first 6 time points; (3) slice timing; (4) realignment; (5) reorient T1; (6) bet; (7) segment; (8) normalize by using T1 image unified segmentation. The detailed steps about preprocessing were presented in our previous studies (Figure 1; Chen et al., 2021; Liu S. et al., 2021; Xu et al., 2021). Subjects with head-motion > 2.0 mm or rotation was > 2.0° were excluded in this study.

### 2.3. Calculation of fALFF and FC

Calculation of fALFF: The procedures included (1) smoothing; (2) detrend and (3) nuisance covariates regression including Friston-24 head motion parameters, global signal, white matter signal and cerebrospinal fluid (CSF) signal. To obtain the power spectrum, the time series of each voxel were transformed to the frequency domain by a fast Fourier transform. The square root was calculated at each frequency of the power spectrum. A ratio of the power spectrum of the low-frequency range (0.01–0.08 Hz) to that of the entire frequency range was calculated, which was regarded as fALFF. Finally, the fALFF value was standardized using Fisher’s r-to-z transformation (zfALFF), which could improve the normality. The details about calculation of fALFF values were presented in previous studies (Zou et al., 2008; Liu S. et al., 2021).

Calculation of FC: The procedures included (1) smoothing; (2) detrend; (3) band pass filter (0.01–0.08 Hz), and (4) nuisance covariates regression including Friston-24 head motion parameters, global signal, white matter signal and cerebrospinal fluid (CSF) signal. Then the brain regions with changed fALFF values were selected as regions of interest (ROI), and FC values between ROIs and the whole brain regions were calculated. *Pearson’s* correlation coefficients between ROIs and the whole brain regions represented the strength of the FC values. The correlation coefficients were converted to z-scores with Fisher’s r-to-z transform for further analysis.

### 2.4. Statistical analysis

Two sample *t*-tests were used to compare the differences of demographic and clinical data between groups using the SPSS software. *P* < 0.05 was considered statistically significant differences. In addition, two sample *t*-tests (two tailed) were performed to compare the differences of fALFF and FC values between groups with age and year of education as covariates by the REST software (Xu et al., 2021). The significant differences were set at voxel *P* < 0.001 and cluster *P* < 0.05 (corrected by gaussian random field, GRF).

## 3. Results

### 3.1. Demographic and clinical characteristics of pED and HCs

There were no differences in the age and educational level between groups. However, pED patients had decreased IIEF-5 scores when compared with HCs. In addition, pED patients had increased ASEX and STAI-S scores when compared with HCs. The details about demographic and clinical characteristics were presented in Table 1.

**TABLE 1:** Demographic and clinical characteristics of pED and HCs.

Variables	pED (*n* = 31)	HCs (*n* = 31)	*P*-value
Age (years)	30.35 ± 5.44	31.94 ± 7.73	0.35
Educational level (years)	14.03 ± 3.11	14.16 ± 1.75	0.85
IIEF-5	11.61 ± 5.02	22.81 ± 0.75	<0.01
ASEX	19.10 ± 3.45	6.90 ± 1.51	<0.01
STAI-S	45.87 ± 10.81	35.84 ± 5.84	<0.01

pED, psychological erectile dysfunction; HCs, healthy controls; IIEF-5, International Index of Erectile Function; ASEX, Arizona Sexual Scale; STAI-S, State-Trait Anxiety Inventory.

### 3.2. Differences of fALFF values between pED and HCs

**FIGURE 2:**
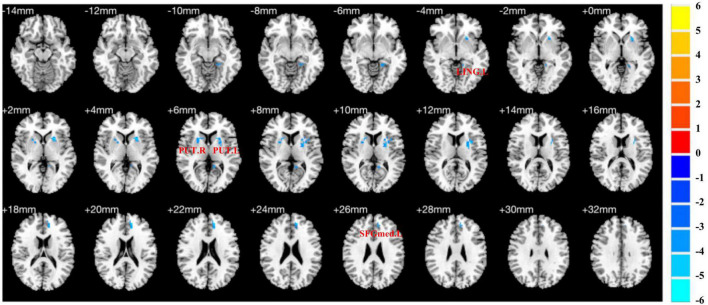
Brain regions show differences in the fALFF values between pED and HCs. pED, psychological erectile dysfunction; HCs, healthy controls; fALFF, fractional amplitude of low-frequency fluctuation; SFGmed.L, left medial superior frontal gyrus; LING.L, left lingual gyrus; PUT.L, left putamen; PUT.R, right putamen.

Compared to HCs, pED patients demonstrated decreased fALFF values in the left medial superior frontal gyrus, lingual gyrus and bilateral putamen (*P* < 0.05) (Table 2; Figure 2).

**TABLE 2:** Differences of fALFF values between pED and HCs.

Brain regions	MNI coordinate			Cluster size	Peak *t*-value
	***X***	***Y***	***Z***		
SFGmed.L	−9	42	27	26	−5.47
LING.L	−15	−42	−3	63	−6.60
PUT.L	−21	15	3	64	−6.38
PUT.R	21	15	6	17	−5.16

pED, psychological erectile dysfunction; HCs, healthy controls; fALFF, fractional amplitude of low-frequency fluctuation. X, Y, and Z: coordinates of peak voxels of the clusters in the montreal neurological institute (MNI) space. SFGmed.L, left medial superior frontal gyrus; LING.L, left lingual gyrus; PUT.L, left putamen; PUT.R, right putamen.

### 3.3. Differences of FC values between pED and HCs

**FIGURE 3:**
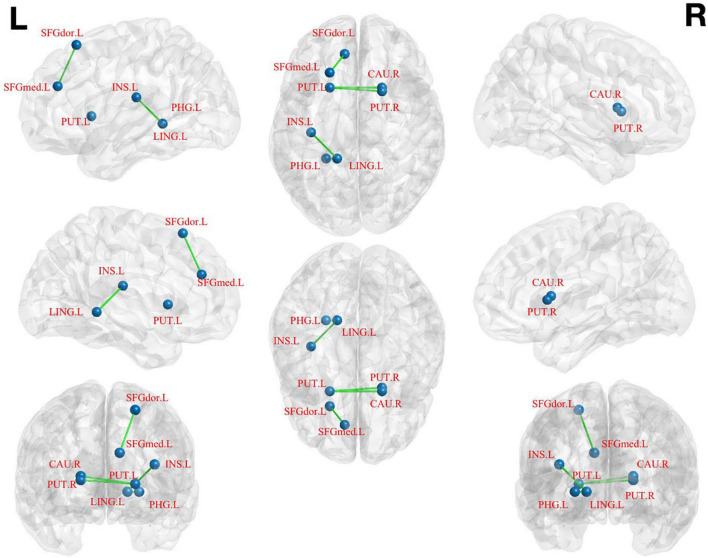
Brain regions show differences in the FC values between pED and HCs. pED, psychological erectile dysfunction; HCs, healthy controls; FC, functional connectivity; SFGdor.L, left dorsolateral superior frontal gyrus; SFGmed.L, left medial superior frontal gyrus; INS.L, left insula; PHG.L, left parahippocamal gyrus; LING.L, left lingual gyrus; CAU.R, right caudate; PUT.L, left putamen; PUT.R, right putamen.

(1) The left medial superior frontal gyrus as ROI: pED patients showed decreased FC values in the left dorsolateral superior frontal gyrus when compared with HCs (Table 3; Figure 3).

**TABLE 3:** Differences of FC values between pED and HCs.

ROI	Brain regions	MNI coordinate			Cluster size	Peak *t*-values
		***X***	***Y***	***Z***		
SFGmed.L	SFGdor.L	−21	27	60	45	−5.45
LING.L	PHG.L	−24	−42	−3	181	−8.03
	INS.L	−36	−21	18	138	−5.61
PUT.L	CAU.R	21	12	9	101	−6.02
PUT.R	PUT.L	−27	3	12	195	−6.59
	CAU.R	21	9	12	191	−6.28

pED, psychological erectile dysfunction; HCs, healthy controls; FC, functional connectivity; ROI, regions of interest. X, Y, and Z: coordinates of peak voxels of the clusters in the montreal neurological institute (MNI) space. SFGmed.L, left medial superior frontal gyrus; SFGdor.L, left dorsolateral superior frontal gyrus; LING.L, left lingual gyrus; PHG.L, left parahippocamal gyrus; INS.L, left insula; PUT.L, left putamen; PUT.R, right putamen; CAU.R, right caudate.

(2) The left lingual gyrus as ROI: decreased FC values were found in the left parahippocamal gyrus and insula of pED patients (Table 3; Figure 3).
(3) The left putamen as ROI: decreased FC values were identified in the right caudate of pED patients (Table 3; Figure 3).
(4) The right putamen as ROI: pED patients had decreased FC values in the left putamen and the right caudate when compared with HCs (Table 3; Figure 3).

### 3.4. Correlations between altered brain regions and clinical characteristics

**FIGURE 4:**
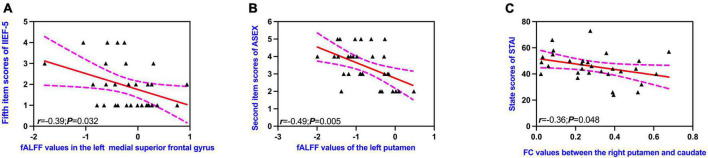
Correlations between altered brain regions and clinical characteristics **(A–C)**. IIEF-5, International Index of Erectile Function; ASEX, Arizona Sexual Scale; STAI-S, State-Trait Anxiety Inventory; fALFF, fractional amplitude of low-frequency fluctuation; FC, functional connectivity. Dotted lines indicated 95% confidence lines. The significant level was set at *P* < 0.05 (uncorrected).

The fALFF values of the left medial superior frontal gyrus were negatively correlated with the fifth item scores of IIEF-5 (Figure 4), as well as the second item scores of ASEX in patients with pED (Figure 4) (uncorrected). FC values between the right putamen and caudate showed negative correlations with the state scores of STAI-S in patients with pED (Figure 4) (uncorrected).

## 4. Discussion

In this study, rs-fMRI data of pED patients were collected and analyzed to explore the changes of brain function and their relationships with clinical characteristics of patients. The results showed that pED patients had decreased fALFF and FC values in the superior frontal gyrus and caudate-putamen, which were related to the sexual function and psychological condition of patients. However, the results of the correlation analyses did not survive correction for multiple comparisons. Further studies with larger sample sizes were needed to improve the statistical power and to verify these findings. These altered brain regions might be involved in the central pathological mechanisms of pED and related to the clinical features including ED and abnormal psychosocial status.

Putamen is one of the most important structures composed of the basal ganglia-thalamic-cortex circuit and communicates with limbic regions, such as cingulate cortex, hippocampus and amygdala, which play an important role in the regulation of cognition and emotion (Peters et al., 2016). Previous study had found that the putamen was activated by sexual arousal and sexual stimulation while the striatum was activated obviously when the penis was erectile (Arnow et al., 2002). In addition, dopamine is projected from the medial hypothalamus and substantia nigra to the hypothalamus and striatum respectively, and dopamine agonists can promote penile erection and improved erection hardness (Arnow et al., 2002). Considering the important role of putamen in the dopamine circuit, negative associations between fALFF values of the left putamen and the second item scores of ASEX might suggest that impaired brain activity of this brain region might lead to the difficult of sexual arousal of pED patients.

The prefrontal cortex plays an important role in sexual behavior, as well as cognition and emotion (Spinella, 2007). As part of the emotion regulation system, the prefrontal cortex involves in the top-down regulation of emotion and attention (Disner et al., 2011). At the same time, it projects onto the ventral tegmental area (VTA) and the nucleus accumbens, which are generally considered to be major components of the brain’s reward system (Tzschentke, 2000). The medial prefrontal cortex is part of the dopaminergic system and it receives prominent dopaminergic input from VTA and input from other subcortical structures *via* the medial thalamus. Considering the important role of the medial prefrontal cortex in the reward circuit, negative relationships between fALFF values of the left medial superior frontal gyrus and the fifth item scores of IIEF-5 might suggest that the functional impairment of this brain area might be related to the lower sexual satisfaction of patients. The dysfunction of the left medial superior frontal gyrus might lead disability to obtain pleasure from sexual behavior.

The prefrontal system has connections with many other brain regions, which are associated with the regulation of sexual behavior, as well as the regulation of cognition, emotion and behavior (Spinella, 2007). The dysfunction of superior frontal gyrus was found to be involved in the occur of mood disorders (Zhao et al., 2019). The medial prefrontal cortex plays a vital role in the emotional self-regulation (Ozawa et al., 2019; Yankouskaya and Sui, 2021). In healthy subjects, inactivation of the medial prefrontal lobe during visual stimulation was found to be negatively correlated with erectile response (Moulier et al., 2006). This brain region’s activation is linked to general arousal, self-relevance of visual stimulation, which is involved in the erectile response. As a result, pED patients might pay less attention to the level of sexual arousal and derive less pleasure from visual stimulation. Furthermore, there were evidences that abnormalities of the medial prefrontal cortex caused advanced processing deficits in pED patients in response to sexual stimuli, as well as feedback from physiological changes (Cera et al., 2014).

In this study, as a part of the dorsolateral prefrontal lobe, decreased FC of the left dorsolateral superior frontal gyrus was consistent with previous findings, and activity of the left dorsolateral prefrontal lobe was positively correlated with sexual performance and sexual satisfaction (Cera et al., 2014). The dorsolateral prefrontal lobe is primarily responsible for cognitive control, response inhibition, behavioral flexibility, attention, and long-term planning (Cheng et al., 2015). Patients with pED were found to have abnormal inhibition of the dorsolateral prefrontal cortex, which impaired their perception and attention to sexual objects (Yin et al., 2020). Previous study had found that FC values of the dorsolateral prefrontal cortex was inversely associated with the severity of anxiety (Ball et al., 2013), and the dorsolateral prefrontal cortex had a top-down modulatory effect on attentional bia in these anxious patients (Heeren et al., 2017). Therefore, in this study, pED patients with decreased FC between the left medial superior frontal gyrus and dorsolateral superior frontal gyrus, might have difficulties with self-emotional regulation and memory repair of past memories in the context of sexual behavior. The impairment of concentration might result in the inability to fully engage in sexual activity and the inability to pay attention to their own pleasure and feelings. This problem might further lead to the fail to obtain sexual pleasure, which might also account for the difficulties in sexual arousal and the reduced ability to obtain pleasure from visual sexual stimuli in pED patients (Cera et al., 2014).

Anatomically, the striatum is the major component of the basal ganglia, including the putamen and caudate. The putamen and caudate are connected to each other by a large number of connections. In previous study, it was found that the striatum (putamen and caudate) received various afferent fibers from the limbic system (Liu X. et al., 2021), and the ventral striatum belonged to the limbic system (Yoo et al., 2019). The decreased FC between the left putamen and right caudate might lead to functional abnormalities of the limbic system, particularly on the left side of the brain, which might cause the inability to control or regulate anxiety and inhibit sexual activity during sexual activity. Previous studies had found that pED patients were more likely to suffer from depression and anxiety than healthy individuals, which might be related to the decreased FC of brain regions in the limbic system (Chen et al., 2017; Lu et al., 2021).

Finally, putamen and caudate nucleus, as the core of basal ganglia, form the basal ganglia-thalamic-cortex circuit with thalamus, and the weakened connection is associated with the ability to regulate emotion (Spinella, 2007). As the important components of the reward circuit, the putamen and caudate were active during sexual arousal, and dopamine secretion from this circuit was also beneficial to penile erection (Arnow et al., 2002). In this study, FC values between the right putamen and caudate were negatively related to the scores of STAI-S, which suggested that the decreased FC values between these regions might be related to the anxious emotion of pED patients. Decreased FC values of these brain region might be responsible for the difficulty in overcoming the fear of the experience of failed sexual behaviors and the regulation of anxiety during sexual behaviors in pED patients.

## 5. Conclusion

In conclusion, our results suggested that pED patients had brain regions with altered activities within the medial superior frontal gyrus and caudate-putamen, which were associated with sexual function and psychological condition. This study also investigated the decreased FC values of these brain region, which were the core of basal ganglia-thalamic-cortex and important components of the reward circuit. These findings provided new insights into the central pathological mechanisms of pED.

## Data availability statement

The raw data supporting the conclusions of this article will be made available by the authors, without undue reservation.

## Ethics statement

The studies involving human participants were reviewed and approved by Jiangsu Province Hospital of Chinese Medicine, Affiliated Hospital of Nanjing University of Chinese Medicine, Nanjing, China. The patients/participants provided their written informed consent to participate in this study.

## Author contributions

JC and YC designed the experiments. XL, SL, TL, and JC analyzed the results and wrote the manuscript. All authors contributed to clinical data collection and assessment and approved the final manuscript.
